# Case Report: Pazopanib-induced acute coronary syndrome

**DOI:** 10.3389/fcvm.2024.1466395

**Published:** 2024-09-24

**Authors:** Adithya K. Yadalam, William M. Schultz, Chanhee Han, Anant Mandawat

**Affiliations:** ^1^ Division of Cardiology, Department of Medicine, Emory University School of Medicine, Atlanta, GA, United States; ^2^ Division of Gynecologic Oncology, Department of Gynecology and Obstetrics, Emory University School of Medicine, Atlanta, GA, United States; ^3^ Winship Cancer Institute of Emory University, Atlanta, GA, United States

**Keywords:** pazopanib, acute coronary syndrome, cardio-oncology, interventional cardiology, case report, coronary artery disease

## Abstract

**Introduction:**

Pazopanib is a tyrosine kinase inhibitor approved for the treatment of metastatic renal cell carcinoma and advanced soft-tissue sarcoma that functions by inhibiting vascular endothelial growth factor receptors. Although the package insert and current cardio-oncology guidelines indicate a risk of acute coronary syndrome (ACS) associated with pazopanib, the causative role of pazopanib in arterial thrombosis is unclear due to a lack of focused coronary disease evaluation in oncological clinical trials prior to pazopanib initiation. Herein we present an antecedent ischemic evaluation of a patient who was prescribed pazopanib to demonstrate the first reported case of ACS directly attributable to pazopanib.

**Case description:**

A 65-year-old woman with metastatic leiomyosarcoma presented to the hospital with ACS. Pazopanib had been initiated 8 months prior, and an ischemic evaluation 6 weeks prior to hospitalization indicated mild coronary artery disease (CAD). Emergent cardiac catheterization revealed a large thrombotic occlusion of the mid-left anterior descending coronary artery involving the secondary diagonal artery, which was treated with manual aspiration thrombectomy. Pazopanib was discontinued, and the patient was discharged from the hospital 12 days later.

**Discussion:**

Although pazopanib is associated with ACS, there is a lack of definitive data supporting this association. This case-based demonstration of pazopanib-induced ACS provides a discrete clinical example of this phenomenon. The patient's minimal atherosclerotic burden 6 weeks prior to her presentation for ACS strongly suggests causality attributable to pazopanib. Given the increased risk for ischemic heart disease, careful attention and an individualized risk assessment for CAD should be provided to patients who are prescribed pazopanib.

## Introduction

Pazopanib is a tyrosine kinase inhibitor (TKI) approved for the treatment of metastatic renal cell carcinoma and advanced soft-tissue sarcoma (STS) and functions by inhibiting vascular endothelial growth factor (VEGF) receptors (1). Although current European Society of Cardiology (ESC) Cardio-Oncology guidelines classify pazopanib as being associated with a risk for acute coronary syndrome (ACS), evidence is limited and without clear causation (2–7, 9). Herein we present the first reported case of pazopanib-induced ACS in a patient with metastatic STS.

## Case description

**Figure 1:**
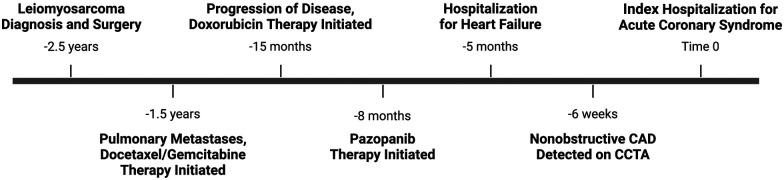
Timeline of clinical events. Depiction of important clinical events in chronological order, beginning with diagnosis of leiomyosarcoma and ending with index hospitalization for acute coronary syndrome.

**Figure 2:**
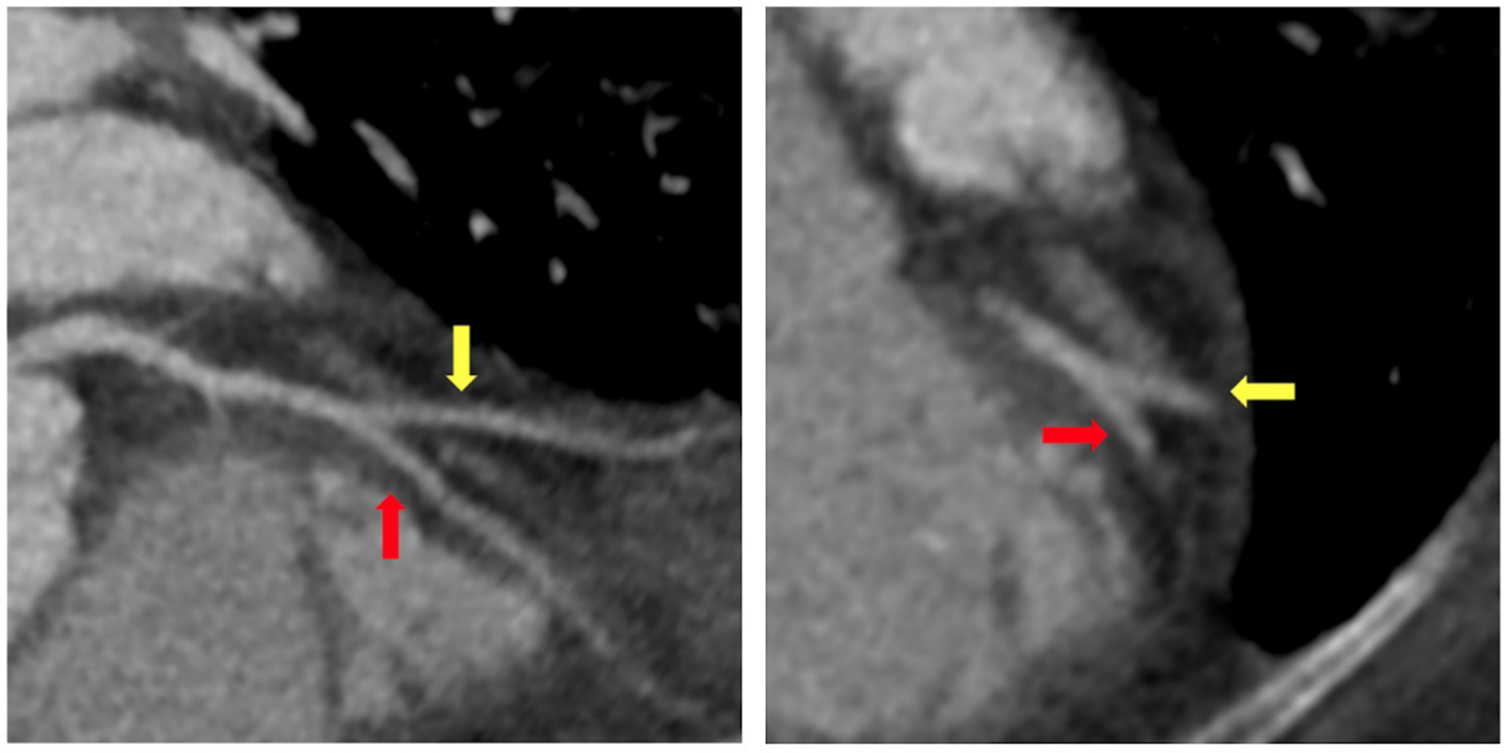
Coronary computed tomography angiography. Coronary computed tomography angiography performed 6 weeks prior to index hospitalization for acute coronary syndrome demonstrated minimal coronary atherosclerosis at the bifurcation of the mid-left anterior descending artery (red arrow) and secondary diagonal artery (yellow arrow).

A 65-year-old woman with a past medical history of hypertension and obesity and without any family history of coronary artery disease (CAD) was diagnosed with Stage IB leiomyosarcoma 2.5 years prior to the index hospitalization for ACS (Figure 1). Her leiomyosarcoma was initially treated with total abdominal hysterectomy, bilateral salpingo-oophorectomy, and bilateral pelvic lymph node dissection. Pathological examination confirmed negative margins and lymph nodes. Recurrent pulmonary metastatic disease was detected 1 year later and treated with docetaxel/gemcitabine. Due to progression of disease despite therapy, doxorubicin was initiated as a second-line treatment 15 months prior to the index hospitalization. An asymptomatic decrease in left ventricular ejection fraction (LVEF) from 60% to 40% with global hypokinesis was observed following doxorubicin therapy, and thus carvedilol was initiated. After a favorable response to a total anthracycline lifetime dose of 508 mg/m^2^, pazopanib was initiated 8 months prior to the index hospitalization due to the lifetime dose limitations of doxorubicin. Carvedilol was switched to metoprolol succinate at the time of pazopanib initiation, given the adverse drug–drug interaction between permeability glycoprotein (P-gp) inhibitors, such as carvedilol, and pazopanib, which can result in increased serum exposure to pazopanib (1). Due to persistently reduced LVEF on repeat evaluation, an ischemic evaluation with coronary computed tomography angiography (CCTA) was performed 6 weeks prior to the ACS admission. The CCTA revealed patent coronary arteries and <25% stenosis of the proximal left anterior descending artery (LAD) (Figure 2). The total coronary artery calcium score was 4. Six weeks later, the patient presented for the index ACS hospitalization.

**Figure 3:**
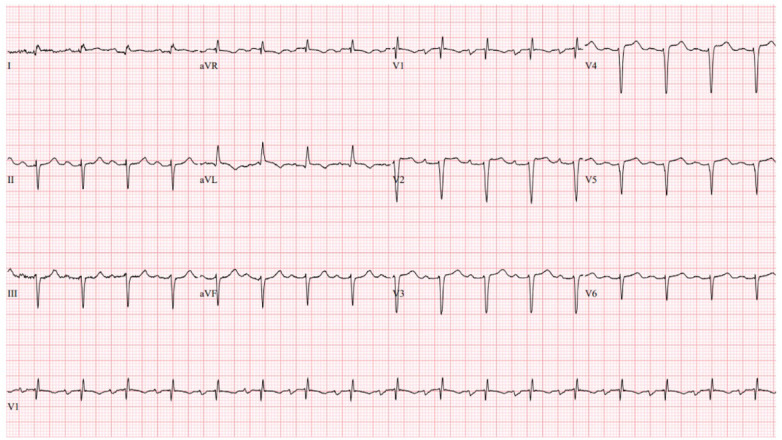
Index hospitalization electrocardiogram. The index hospitalization electrocardiogram demonstrated sinus tachycardia (HR 103 beats per minute), left anterior fascicular block, an RSR’ pattern in V_1_, and non-specific T-wave changes.

**Figure 4:**
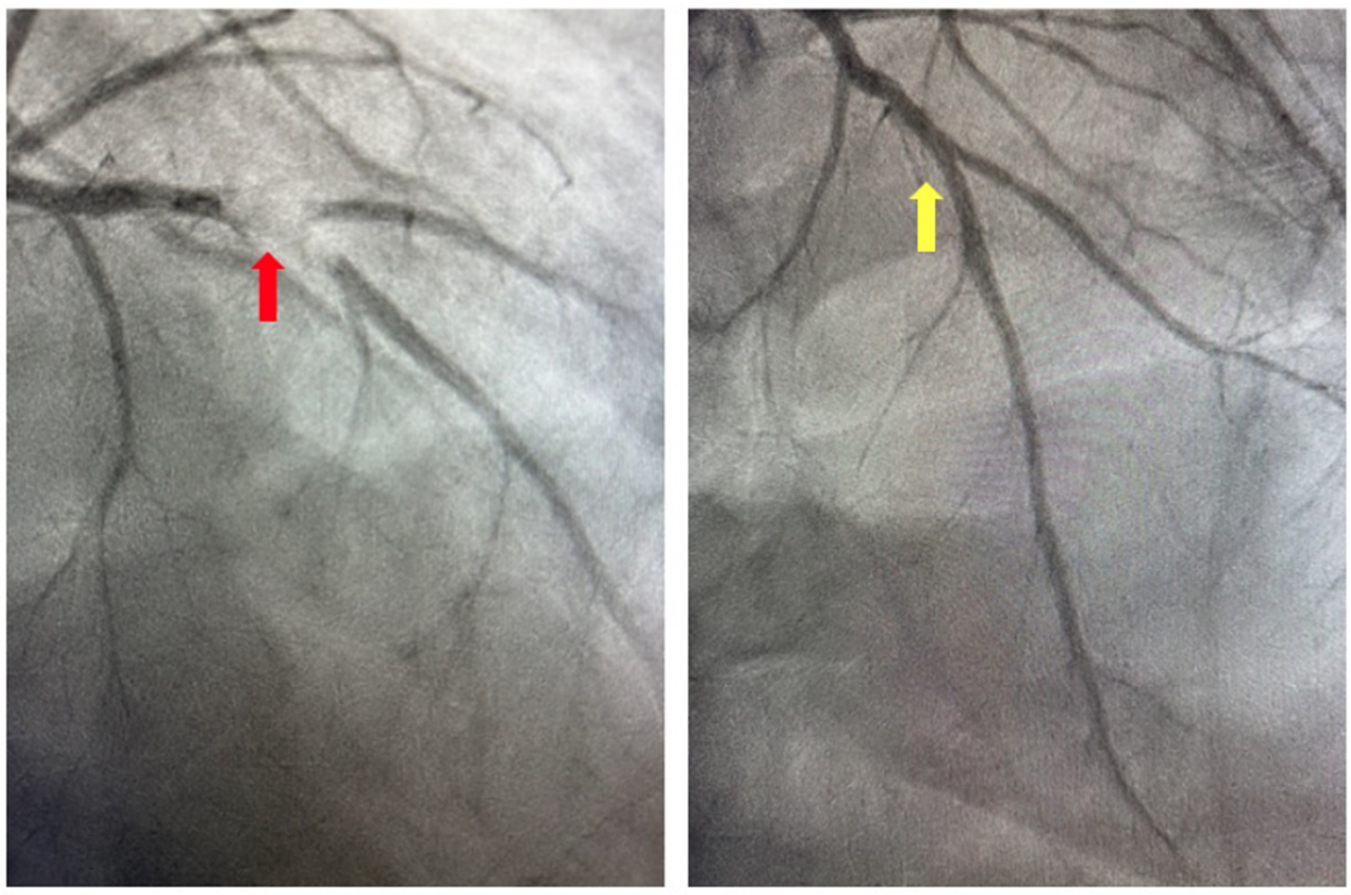
Coronary angiogram. Invasive coronary angiography demonstrating thrombotic occlusion of the mid-left anterior descending artery and second diagonal artery prior to manual aspiration thrombectomy (red arrow) and following manual aspiration thrombectomy (yellow arrow).

On presentation to the index hospitalization, the patient reported nausea, vomiting, and decreased appetite for 2 days. She denied a history of alcohol, tobacco, or recreational drug use. Physical examination demonstrated appropriate mentation, regular tachycardia without abnormal heart sounds, vesicular breath sounds, and no peripheral edema. An electrocardiogram showed sinus tachycardia with a heart rate of 103 beats per minute (Figure 3). High-sensitivity troponin-I was >25,000 ng/L (*ref*. <14 ng/L), and the lactate level was 3.4 mmol/L (*ref*. 0.5–2.2 mmol/L). The most recent prior measurement of high-sensitivity troponin-I was 147 ng/L 4 months prior. Liver enzyme and creatinine levels were increased from baseline. Transthoracic echocardiography (TTE) revealed a decrease in LVEF to 10%, left ventricular internal end-diastolic diameter (LVIDd) of 6.2 cm, severe global hypokinesis, and regional wall motion abnormalities within the LAD distribution. No left ventricular thrombus was observed. Pazopanib and carvedilol were held. Emergent cardiac catheterization revealed a large thrombotic occlusion of the mid-LAD involving the secondary diagonal artery, which was treated with manual aspiration thrombectomy (Figure 4). Right heart catheterization revealed elevated right- and left-sided filling pressures and a decreased cardiac index (Fick) of 1.36 L/min/m^2^. Her hemodynamic status improved following thrombectomy. The patient’s hospital course was complicated by the development of atrial fibrillation with rapid ventricular response, which improved with amiodarone. As the patient's cancer treatment was now limited to a fourth-line therapy with minimal chance of a favorable response, symptom control and supportive therapy were recommended. Pazopanib was discontinued, and the patient was discharged from the hospital 12 days after presentation with a slight improvement in LVEF to 15%–20%. Her discharge medications included aspirin and atorvastatin following her non-ST-elevation myocardial infarction (MI); milrinone, spironolactone, and torsemide for advanced chronic systolic heart failure; and amiodarone and apixaban for atrial fibrillation. The patient was re-hospitalized 3 months later for acute decompensated heart failure following outpatient reduction of her home diuretic dose. During this hospitalization, LVEF was found to have improved to 20%–25%. Upon discharge, her previous home diuretic dose was restored. During post-hospitalization follow-up 1 month later, the patient reported that her heart failure-related symptoms and functional status had improved. Her milrinone dose remains at a stable dose, and she continues to tolerate her regimen of spironolactone, torsemide, amiodarone, and apixaban without issue.

## Discussion

The above case represents, to our knowledge, the first causative report of pazopanib-induced ACS in a patient with metastatic STS. Although current ESC guidelines estimate a 1%–10% risk of ACS associated with pazopanib, clinical trials investigating the safety of pazopanib have not convincingly demonstrated a significant association between pazopanib therapy and ACS due to a lack of focused coronary disease evaluation in oncological clinical trials prior to pazopanib initiation (2–7). A single-arm monotherapy trial of pazopanib in patients with metastatic STS previously observed a 3% (*N* = 1/33) incidence of myocardial infarction incidence; however, the lack of a comparator placebo group makes this finding difficult to contextualize (4). Subsequent randomized, double-blinded, placebo-controlled clinical trials investigating the safety and efficacy of pazopanib in patients with metastatic STS have observed either no ACS events or an MI or ischemia incidence of 2% (*N* = 2/240) in the pazopanib group vs. 0% in the placebo group (Table 1) (5–7). Until now, a case demonstrating a distinct, causal relationship between pazopanib therapy and incident ACS has yet to be described.

**Table 1:** Summary of existing evidence regarding pazopanib-associated acute coronary syndrome.

Reference	Study population	Evaluation for coronary artery disease	Results related to ACS
Lim et al. (2010) (4)	*N* = 33 patients with metastatic soft tissue sarcoma	No focused coronary evaluation; excluded patients with a history of MI, PCI ≤12 weeks from enrollment, or hospital admission for UA	MI occurred in 3% (*N* = 1/33) of pazopanib-treatment patients; no placebo arm available for comparison
Sternberg et al. (2010) (9)	*N* = 435 patients with renal cell carcinoma	No focused coronary evaluation; excluded patients with a history of MI, PCI, or UA	MI or ischemia occurred in 2% (*N* = 5/290) of pazopanib-treated patients compared to no events in placebo-treated patients
Van der Graaf et al. (2012) (5)	*N* = 372 patients with soft tissue sarcoma	No focused coronary evaluation; excluded patients with a history of PCI, MI, UA, or CABG ≤6 months from enrollment	MI or ischemia occurred in 2% (*N* = 2/240) of pazopanib-treated patients compared to no events in placebo-treated patients

ACS, acute coronary syndrome; CABG, coronary artery bypass graft; MI, myocardial infarction; PCI, percutaneous coronary intervention; UA, unstable angina.

Regarding the above case, the patient's patent coronary arteries noted on CCTA 6 weeks prior to her presentation for ACS strongly suggest causality attributable to pazopanib. Moreover, the lack of uncontrolled traditional cardiovascular risk factors or alternative etiologies for ACS further supports this to be a case of pazopanib-induced ACS. Although pazopanib therapy has also been associated with heart failure, the patient's decline in LVEF is substantially more likely a result of anthracycline-induced cardiomyopathy rather than pazopanib, given the temporal relationship with the doxorubicin therapy.

There are several proposed mechanisms for pazopanib-induced thrombosis and myocardial injury. VEGF has been linked to endothelial cell survival and proliferation, as well as the promotion of coronary angiogenesis. Given pazopanib's role in VEGF receptor inhibition, endothelial cell apoptosis and resultant thromboembolic and/or ischemic events are plausible. In addition, pazopanib-induced inhibition of VEGF can create a pro-thrombotic environment through the overproduction of erythropoietin and increased blood viscosity (8).

Although pazopanib use has been associated with ACS, evidence to support this association is lacking. This case provides a discrete example of pazopanib-induced ACS in a patient with non-obstructive CAD demonstrated 6 weeks prior to ACS presentation. Careful attention and an individualized risk assessment for CAD should be provided to patients who are prescribed pazopanib, and pazopanib should be permanently discontinued in patients who experience an arterial thromboembolic event. Future studies should examine whether a dose-response relationship exists between pazopanib therapy and adverse cardiovascular events.

## Data Availability

The original contributions presented in the study are included in the article/Supplementary Material, further inquiries can be directed to the corresponding author.
